# Fine mapping genetic associations between the HLA region and extremely high intelligence

**DOI:** 10.1038/srep41182

**Published:** 2017-01-24

**Authors:** Delilah Zabaneh, Eva Krapohl, Michael A. Simpson, Mike B. Miller, William G. Iacono, Matt McGue, Martha Putallaz, David Lubinski, Robert Plomin, Gerome Breen

**Affiliations:** 1 MRC Social, Genetic and Developmental Psychiatry Centre, Institute of Psychiatry, Psychology & Neuroscience, King’s College London, London SE5 8AF, UK; 2 Division of Genetics and Molecular Medicine, Guy’s Hospital, Great Maze Pond, London, SE1 9RT, UK; 3 Department of Psychology, University of Minnesota, Minneapolis, MN 55455, USA; 4 Duke University Talent Identification Program, Duke University, Durham, NC 27701, USA; 5 Department of Psychology and Human Development, Vanderbilt University, Nashville, TN 37203, USA

## Abstract

General cognitive ability (intelligence) is one of the most heritable behavioural traits and most predictive of socially important outcomes and health. We hypothesized that some of the missing heritability of IQ might lie hidden in the human leukocyte antigen (HLA) region, which plays a critical role in many diseases and traits but is not well tagged in conventional GWAS. Using a uniquely powered design, we investigated whether fine-mapping of the HLA region could narrow the missing heritability gap. Our case-control design included 1,393 cases with extremely high intelligence scores (top 0.0003 of the population equivalent to IQ > 147) and 3,253 unselected population controls. We imputed variants in 200 genes across the HLA region, one SNP (rs444921) reached our criterion for study-wide significance. SNP-based heritability of the HLA variants was small and not significant (h^2^ = 0.3%, SE = 0.2%). A polygenic score from the case-control genetic association analysis of SNPs in the HLA region did not significantly predict individual differences in intelligence in an independent unselected sample. We conclude that although genetic variation in the HLA region is important to the aetiology of many disorders, it does not appear to be hiding much of the missing heritability of intelligence.

Intelligence is correlated with a wide range of important life outcomes including health, education, occupation and income^1^,^2^. The general factor called intelligence accounts for about 40 percent of the total variance when a battery of diverse cognitive tests is administered to a sample with a good range of cognitive ability^3^. Intelligence has consistently been shown to be about 50% heritable in scores of twin and adoption studies^4^ and about 30% in SNP-based heritability estimates^5^,^6^. However, attempts to identify DNA variants responsible for heritability have had limited success, similar to other highly polygenic complex traits and common disorders. The largest genome-wide association (GWA) meta-analysis of more than 50,000 adults reported 13 genome-wide significant SNP associations^6^. However, a polygenic score based on these GWA results accounted for only 1.2% of the variance in independent samples. The most powerful polygenic score to predict intelligence comes from a GWA meta-analysis of a ‘proxy’ variable of years of schooling, which correlates about 0.50 with intelligence. A polygenic score from this GWA analysis of more than 300,000 individuals predicted 3.2% of the variance in years of education in an independent sample^7^. However, 3.2% is less than 10% of the 50% heritability of intelligence. The problem of “missing heritability” is pandemic for complex traits like intelligence as well as common disorders^8^.

In this work we have used imputation and genetic association in an attempt to identify variants in the human leukocyte antigen (HLA) region that account for some of the missing heritability. The major histocompatibility complex (MHC) plays a critical role in many diseases and phenotypes^9^. In humans, the MHC is also called human leucocyte antigen (HLA) and lies on the short arm of chromosome 6. The classical HLA region is approximately 3.37 Mb (HG19: chr6: 29691116–33054976). The HLA gene family provides instructions for making human leukocyte antigen (HLA) complex proteins and consists of more than 200 genes located close together on chromosome 6. The HLA complex includes three basic groups of genes: class I, class II, and class III. HLA molecules present antigenic peptides to generate immune defence reactions. The HLA genes are characterized by extraordinary polymorphism with >1,980 unique known alleles that differ in frequency among different human populations^10^. Whereas HLA class I and class II genes encode sets of structurally related, highly polymorphic transplantation antigens, the class III genes display a low to moderate degree of genetic variability. The proteins produced from the class III genes have different functions; they are involved in inflammation and other immune system activities^10^.

It has been shown that function and volume of the hippocampus and thalamus, regions whose roles include memory consolidation and sensory processing are affected by HLA genes^11^. Variants within the HLA region have been reported to be associated with language impairment^12^, cognitive decline in later life^13^ and mental health phenotypes^14^. However, genetic associations between HLA variants and intelligence have not yet been investigated. Two non-genome wide studies investigated the association between HLA-DRB1 genotypes and cognitive traits in elderly individuals. The first study^15^ investigated aspirin use and genotypes at DRB1*01 and DRB1*05 locus and their results suggested that aspirin use and certain genotypes may influence cognitive traits in non-demented elderly subjects. Another study^16^ also investigating the role of DRB1 alleles and cognitive phenotypes, observed that both DRB1*08 and DRB1*11 were significantly associated with vocabulary ability (cross-sectional and longitudinal scores).

Here we investigate the association between variants in 200 genes across the Class I and II HLA region using a unique case-control design. The cases were 1,393 individuals with extremely high IQ scores (IQ > 147) from the top 0.0003 of the normal distribution of IQ scores. Controls included 3,253 individuals representative of the normal distribution. Cases and controls were from European ancestry from the US. We previously applied this design and sample to conduct a case-control genome-wide analysis of extremely high intelligence using putative functional and exonic variants on the Illumina Infinium HumanExome BeadChip^17^. Despite having 80% power to detect variants that explain >0.0015 of the variation in intelligence for α = 1 × 10^−7^, no individual protein-altering variants were found that were reproducibly associated with extremely high intelligence as well as within the entire distribution of intelligence^17^.

The present study focuses on variants in HLA that are not well tagged in conventional GWAS^18^,^19^. Using the same design, sample and exome array as in our previous study^17^, we investigated whether fine-mapping of the HLA region could narrow the missing heritability gap. The 240 K variants genome-wide on the Illumina Infinium HumanExome BeadChip include 2140 genotyped SNPs variants (MAF > 0.005) in the HLA region, which can be used to impute 6705 HLA variants using a large European reference panel from the Type 1 Diabetes Genetics Consortium [T1DGC]^20^. We aimed to test the association between these variants and extremely high intelligence, comparing allele frequencies for cases consisting of individuals selected for extremely high intelligence scores and controls who were unselected for intelligence.

## Methods

### Sample and genotypes

Data from three samples were used in this study: a sample of high-intelligence cases, a control sample and a representative sample used to extend our case–control results to individual differences in the population. The project received ethical approval from the King’s College London Research Ethics Committee (reference number PNM/11/12–51) and from the European Research Council Executive Agency (reference number Ares (2012)56321). Informed consent was obtained from all subjects. All methods were performed in accordance with relevant guidelines and regulations.

### High intelligence case-control study samples

The 1,409 high cognitive ability sample and 3,253 controls^17^,^21^,^22^ were typed on the Infinium HumanExome BeadChip with a total of 227,858 variants that passed quality control. Further details of the sample, genotyping and quality control are described in ref. 17 and Table 1. The phenotype was measured on the case samples at ages 12 and 13 years old, and the control samples at age 16. All analyses were performed on SNPs that passed quality control (QC) within the chromosome 6 4-Mb HLA region. After QC, we estimated the first 10 principal components using linkage disequilibrium (LD) pruned whole-genome common SNP genotype data from the exome chip to correct for potential population structure in the subsequent analysis that focused on the HLA region only.

### Replication samples

Because no comparison samples of extremely high intelligence are available, we tested the generalization of case-control associations to individual differences in two unselected population samples. If a SNP were associated with extremely high intelligence, we test the prediction that the SNP will also be associated with individual differences in intelligence in the expected direction within the normal distribution of intelligence, a prediction supported by quantitative genetic data^23^. The first unselected sample is our control group of 3,253 individuals for whom intelligence scores were available; associations for individual differences within the control sample should be independent of case-control differences, as we are testing the quantitative variation in these individuals within this sample only. The second unselected sample is the Twins Early Development Study (TEDS^24^ which included 6,710 unrelated individuals (only one member of a twin pair) with cognitive ability (intelligence scores) at age 12.

### Imputation of HLA variants in the exome data

Using the exome data for the High IQ samples and the controls, we extracted SNPs located within the MHC region (chr6: 29–33 Mb on build 37/hg19), removing SNPs with minor allele frequency <0.5%. We used BEAGLE^25^ as implemented in SNP2HLA^19^ to impute all missing SNPs, and checked that alleles in the Hi-IQ genotype file and those in the reference haplotypes refer to the same strand. Both A/T and G/C SNPs were checked to ensure that they aligned to the same strand, by comparing minor allele frequencies, and when these were >0.40 the SNPs were removed. Genotype imputation was conducted for two- and four-digit classical HLA alleles and amino acid polymorphisms of the seven class I and class II HLA genes, as well as the additional SNPs that were not genotyped in the exome data, using SNP2HLA software, and the T1DGC Immunochip/HLA reference panel from data collected from 5,225 unrelated individuals with European ancestry by the Type 1 Diabetes Genetics Consortium (T1DGC)^20^. Genotype data for this reference panel included 7,135 SNPs within the HLA region assayed with the Illumina Immunochip array^26^,^27^. The variants in the reference panel were coded as bi-allelic markers (presence vs. absence), allowing the use of BEAGLE for the imputation. We applied post-imputation quality control criteria: MAF ≥ 5% and imputation score r^2^ ≥ 0.90, for the association analysis. These procedures provide genotypes for 816 classical 2- and 4-digit alleles, and 711 amino acid residues for the 3 class I HLA genes (HLA-A, HLA-B and HLA-C) and 5 class II HLA genes (HLA-DRB1, HLA-DQA1, HLA-DQB1, HLA-DPA1 and HLA-DPB1), where the total number of QCed HLA variants was equal to 5,685, these include the original genotyped 1967 SNPs. A summary of imputed variants is in Table 2, and numbers of classical alleles are in Supplementary Table S1.

### Statistical analysis

#### Power

Assuming an additive model, with a conservative type I error rate α = 1 × 10^−7^ accounting for all 70,112 SNPs from the Illumina HumanExome-12v1-1 Array^17^, and our case-control design where the cases have been selected from 0.0003 of the distribution, we have 70% power to detect associated variants explaining >0.0025 of the trait variance^28^. Power for different effect sizes is summarised in Supplementary Table S2, R^2^ is Nagelkerke’s pseudo-R^29^.

#### Logistic regression

Association analyses were carried out using additive genetic models for all genotyped SNPs, followed by an analysis for the hard-called imputed SNPs and classical alleles in the region using logistic regression as implemented in PLINK1.9^30^,^31^. In the model we included the first 10 principal components, to control for population stratification, and sex as covariates.

### Stepwise conditional analysis

Conditional analysis allows testing all the SNPs in the region (or a chosen subset according to statistical significance) to identify the most significant one, and repeating the analysis conditioned on the most significant SNP to see if others are significant in addition to the top SNP. The process is repeated, each time conditioning on all SNPs that emerged as most significant in prior rounds, until all SNPs whose significance is dependent on other SNPs are identified and removed, this method was suggested for the analysis of the HLA region and genetic data by Cordell and Clayton^32^, and has been used in HLA variants association analysis, for example^33^. These analyses were carried out using R.

### Gene-based association analysis

Gene-based association was carried out using Vegas2^34^,^35^. This software uses algorithms that assign variants to genes using a simulation approach and subsequently calculates the gene-based empirical association p-values. This is done by considering all SNPs within a gene as a unit for the association analysis, and it can be a powerful complement to the single SNP–trait association analysis. After reading the GWAS summary file with SNP IDs (restricted to dbSNPs only), Vegas2 assigns variants to genes based on the hg19 genomic location and using a simulation approach, the gene-based empirical association p-values are then calculated. We performed this gene-based analysis using the P-values generated from the single SNP analysis. As there are ~20,000 genes in the genome, the genome-wide P-value threshold for declaring statistical significance following the Bonferroni correction for multiple testing in the gene-based analyses is 0.05/20,000 = 2.5 × 10^−6^, and for this HLA-region with 200 genes is 0.05/200 = 2.5 × 10^−4^.

### Variance explained

We estimated the proportion of variance in liability to IQ explained by SNPs using Genome-wide Complex Trait Analysis- GCTA^36^. GCTA uses genome-wide SNP genotypes to calculate heritability in the population from identity-by-state relationships for each pair of individuals. In these data only the variants from the HLA region were used. We used hard-called imputed data as described earlier. GCTA calculates the genetic similarity between subjects using all SNPs and uses the restricted maximum likelihood approach to estimate the heritability. We adjusted for sex and the first 10 principal components by including them as covariates in the model. IQ prevalence was estimated to be 0.0003 based on our selection criteria described above, and GCTA used the provided trait prevalence to transform the estimated heritability to the liability scale.

### Polygenic scores

We used genome-wide SNPs and phenotype data from 6,710 unrelated adolescents drawn from the UK-representative TEDS. We processed the 6,710 genotypes using stringent quality control procedures followed by imputation of SNPs using the Haplotype Reference Consortium reference panel, details are in Supplementary text. After quality control, we included around 7,581,516 million genotyped or well-imputed (info ≥ 0.70) variants into the polygenic score analyses. We created genome-wide polygenic scores for each individual in the TEDS sample using summary statistics from the HLA association analysis. PRSice^37^ was used to build a multi-SNP prediction model for the polygenic score analyses. Here polygenic scores are created from the high cognitive ability case-control association results and are used to estimate the proportion of the phenotypic variation in the TEDS sample that is due to genotypic information alone. To do this, PRSice identified independent SNPs from using a P-value informed LD clumping approach as implemented in PLINK with a pairwise r^2^ ≤ 0.25 threshold and a 200-KB window. Independent SNPs, significant at different P-value thresholds are identified and the scores are created weighted by effect size. General linear models were conducted to predict general cognitive ability at age 12 and educational achievement at the end of compulsory education at age 16, using the first 10 principal components as well as genotyping array and plate as covariates to control for population stratification and possible genotyping errors, respectively.

## Results

### Multiple testing

Although 5,685 SNPs were analysed, and a Bonferroni correction for a 0.05 nominal P-value is equal to 8.70 × 10^−6^, due to the very high LD in the region, a suggestive study-wide significance threshold of P = 1.10 × 10^−4^ (0.05/462) was set based on the effective number of tests and a Bonferroni correction: We assumed that SNPs with r^2^ LD < 0.20 were considered independent, and for 5,685 tested variants, 462 independent SNPs were identified from applying a 100 base-pair sliding window with 10 overlapping variants, both thresholds are shown.

### Variance explained

Assuming prevalence = 0.0003^17^, with a rigorous MAF threshold (≥0.05) the variance explained (heritability) on the liability scale from the imputed SNPs (n = 5,685) using GCTA as described above was h^2^ = 0.0028 (SE = 0.0018, P-value = 0.0198).

### Single SNP logistic regression with an additive model

**Figure 1:**
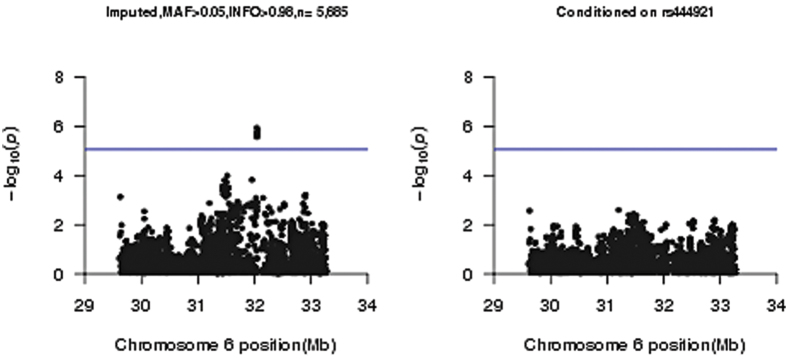
Plots of −log_10_ p-values for association for HLA-region genotyped SNPs and imputed SNPs, (**a**) For the fully adjusted model using all imputed SNPs, (**b**) same as (**a**) conditioning on top SNP rs444921.

**Figure 2:**
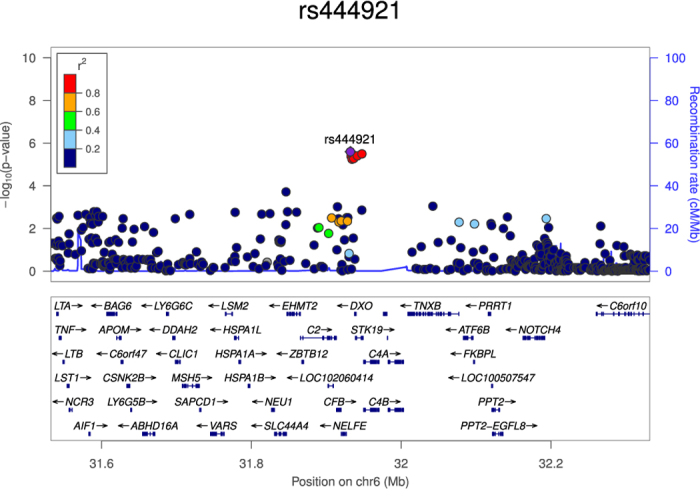
Regional plot for SNP rs444921.

After imputation, we obtained genotypes for two- and four-digit classical alleles, as well as amino acid polymorphisms of the class I and class II HLA genes and SNPs in the HLA region. We carried out a logistic regression assuming an additive effect for all the imputed and genotyped HLA variants, and we observed the top association signal at the genotyped SNP rs444921 (P = 2.53 × 10^−6^). This intronic SNP maps to SKIV2L (Ski2 Like RNA Helicase), a protein coding gene. This SNP is in a highly gene dense region and is in high LD (r^2^ ≥ 0.99) with another 5 SNPs mapping to genes SKIV2L and STK19. All results from the logistic regression for the imputed SNPs are in Fig. 1(a), and a summary description of SNPs with P ≤ 1.0 × 10^−4^ are in Table 3, and P ≤ 1.0 × 10^−3^ in Supplementary Table S3. A regional plot for the top SNP and other SNPs in LD is shown in Fig. 2. A lookup of the association for our top SNPs: rs444921 and rs389512 in the two replication samples and four other studies is in Supplementary Table S4, and a meta-analysis for the top SNP with these studies is illustrated in Supplementary Figure S1. A summary of previous HLA-variant association studies with neuropsychiatric traits is presented in Supplementary Table S5. A lookup of the published associations of DRB1_0801 and DRB1_1101 in our analyses is in Supplementary Table S6.

### Forward stepwise conditional analysis

Using a forward stepwise conditional analysis as described earlier, after conditioning on rs444921, no SNPs were retained in the model, Fig. 1(b).

### Gene-based analysis

**Figure 3:**
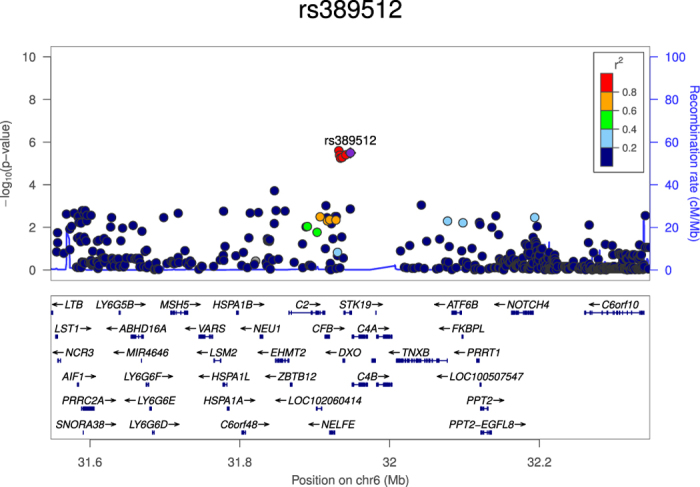
Regional plot for SNP rs389512.

To test whether the aggregate effect of a group of SNPs within a gene is associated with high IQ, we conducted a gene-based analysis using VEGAS2 and found that C4B_2_1 (Complement Component 4B- Chido Blood Group) was the most strongly associated gene with IQ (P = 1.65 × 10^−4^). This gene, is in close proximity to both SKIV2L and STK19 with similar association P-values: P = 1.88 × 10^−4^ with both genes, these results are in Table 4. As is evident from Table 4, the top SNP reported is rs389512 which maps to STK19: Serine/Threonine Kinase 19, and is in high LD with variants in the surrounding genes (Fig. 3). STK19 encodes a serine/threonine kinase which localizes to the nucleus, its specific function is unknown, and it is possible that phosphorylation of this protein is involved in transcriptional regulation (www.genecards.org).

### Polygenic Scores

We investigated evidence for the polygenic nature of extremely high intelligence association with HLA region by building a multi-SNP predictor to predict intelligence and educational attainment in the TEDS data. High-resolution scoring was used from P-value threshold: pT 0 to 1 at 0.0001 intervals in PRSice to find the best-fit polygenic score. There were 4,110 markers from the HLA-TIP data that aligned with the TEDS data. We found no significant correlation between predictors and intelligence or education achievement. The best-fit HLA score for cognitive ability (best-fit-pT = 0.009; number of snps = 19, explained r^2^ = 0.00023 and P = 0.37). The best-fit HLA score for educational achievement (best-fit-pT = 0.0009; number of snps = 6, explained r^2^ = 0.0008 and P = 0.05.

## Discussion

We imputed SNPs across the MHC region as well as classical alleles of HLA genes and their corresponding amino acids and tested all variants for association with high intelligence using a case-control study design, where the extremely high intelligent sample corresponds to cases and is compared to unselected sample controls. Sampling from the extreme of the distribution of a trait can result in a substantial increase in power relative to random sampling with the same number of individuals^38^ because individuals with extremely high IQ should be enriched for IQ-increasing alleles, both rare and common. There are 304 separate studies that report HLA-variant associations with 528 catalogued traits from genome-wide association studies in the GWAS catalogue^39^, of these 9 are neuropsychiatric traits (Supplementary Table S5). Considering the involvement of variants in the HLA region in neuropsychiatric traits, many of which are termed neurodevelopmental disorders, we hypothesised that the HLA might play a functional role in high intelligence.

We did not find any individual HLA SNPs that were associated with extremely high intelligence with the widely accepted genome-wide significance level (P ≤ 5 × 10^−8^). Additionally, Spain *et al*.^17^ had tested the exome chip in their GWAS study, using the same data as our study, where all the functional variants on the array explained 17% of the genetic variance of IQ. In our study, the common imputed and genotyped variants in the HLA region only explained 0.3%. This suggests that the HLA region in our data does not play a sizable role in contributing to the genetic variance of high intelligence, although we were not able to assess an enrichment or lack thereof in HLA as the exome chip has a specific set of common tag SNPs for HLA and, primarily, rare variants elsewhere in the genome.

One sentinel significant SNP: rs444921 mapping to SKIV2L persisted after an HLA-wide Bonferroni correction, P-value = 2.53 × 10^−6^. In our gene-based analysis a second SNP rs389512 also demonstrated a significant association, it should be noted that the two SNPs are in very high LD with (r^2^ = 0.99). When conditioning on the rs444921 SNP, no HLA-wide significant SNPs remain in the model. Although rs444921 shows evidence of association through the single SNP and gene-based tests, there is no strong support for this as genome-wide significance was not established, and our search for replication in six other studies failed to replicate the association (Supplementary Table S4, Supplementary Figure S1). Therefore, overall, the evidence provided by the single-SNP and gene-based analysis is not strong enough to suggests that the MHC region is associated with high IQ.

Two non-genome wide studies investigated the association between HLA-DRB1 genotypes and cognitive traits in elderly individuals, and found significant associations with cognitive traits^13^,^15^. These studies performed a small number of tests and their declaration of significance was less stringent than in our study. The first study by Shepherd *et al*.^15^ incorporated aspirin use in their hypothesis and analyses, which is quite different to our analyses. The second study by Payton *et al*.^13^ have shown that DRB1_0801 and DRB1_1101 were associated with vocabulary ability in a cohort of healthy older white Caucasian volunteers. DRB1_1101 was associated with improved vocabulary, whilst DRB1_0801 was associated with impaired vocabulary ability as well as impaired memory ability. In our study, DRB1_0801 was associated with lower IQ (OR = 0.67, p = 0.01), but significance does not persist after multiple testing (Supplementary Table S6).

The various analyses presented here provide an in-depth investigation of thousands of genotyped and imputed SNPs and classical alleles in the HLA region and, although we identified a possible suggestive association, we were unable to replicate our findings in related phenotypes in independent samples. We conclude that although the HLA region is importantly involved in many diseases, it does not appear to be more than a minor contributor to heritability of intelligence.

## Additional Information

**How to cite this article:** Zabaneh, D. *et al*. Fine mapping genetic associations between the HLA region and extremely high intelligence. *Sci. Rep.* **7**, 41182; doi: 10.1038/srep41182 (2017).

**Publisher's note:** Springer Nature remains neutral with regard to jurisdictional claims in published maps and institutional affiliations.

## Supplementary Material

Supplementary Material

## Figures and Tables

**Table 1:** Summary description of the two data sets that comprised the case-control study.

	n	Mean IQ (SD)	Range
Cases	1,393	181.9 (8.7)	147–222
M	815	182.7 (8.8)	156–219
F	578	180.6 (8.4)	147–222
Controls	3,253	105.5 (14.3)	67–151
M	1,500	105.7 (14.4)	70–151
F	1,753	105.3 (14.2)	67–147

**Table 2:** Counts of the HLA region genotyped and imputed SNPs.

		MAF > 0.0	MAF > 0.005	MAF > 0.01	MAF > 0.05
Genotyped		2,229	2,140	2,121	1,967
Imputed	All variants	8,962	6,705	6,532	5,685
	rs and 1 kg SNPs	4,797	4,789	4,713	4,158
	Classical alleles	3,363	1,130	1,056	816
	Amino acid	802	786	763	711

SNPs are filtered on imputation r^2^ = 0.90, imputed variants include genotyped SNPs.

**Table 3:** Summary results for the adjusted logistic regression model.

SNP	BP	r^2^ with rs444921	Ref allele	N	OR	SE	P	Location	Gene	TIP- MAF	CON-MAF
rs444921	32040156	1.00	A	4662	0.67	0.09	2.53 × 10^−6^	intronic	SKIV2L	0.13	0.13
rs389512	32055573	0.99	G	4662	0.67	0.08	3.20 × 10^−6^	intronic	STK19	0.13	0.13
rs387608	32049536	0.99	T	4662	0.68	0.08	3.96 × 10^−6^	intronic	STK19	0.13	0.13
rs406936	32041140	1.00	T	4662	0.68	0.08	4.14 × 10^−6^	intronic	SKIV2L	0.13	0.13
rs449643	32044658	0.99	A	4662	0.68	0.08	5.29 × 10^−6^	exonic	SKIV2L	0.13	0.13
rs454212	32042351	1.00	A	4662	0.68	0.08	5.66 × 10^−6^	intronic	SKIV2L	0.13	0.13
rs2524276	31516244	—	T	4662	0.60	0.13	7.34 × 10^−5^	intergenic	MICA,HCP5	0.07	0.06

SNPs that reached significance with p < 1.1 × 10^−3^. Logistic model with sex and the first 10 principal components as covariates.

**Table 4:** Gene-based association analysis using Vegas2 for p-values < = 1 × 10^−3^.

Gene	Number of SNPs	P-value	Top SNP	Top SNP p-value
C4B_2_1	14	1.65 × 10^−4^	rs389512	3.20 × 10^−6^
C4B_1	14	1.67 × 10^−4^	rs389512	3.20 × 10^−6^
C4A_1	14	1.76 × 10^−4^	rs389512	3.20 × 10^−6^
LOC102060414	15	1.83 × 10^−4^	rs389512	3.20 × 10^−6^
MIR1236	15	1.85 × 10^−4^	rs389512	3.20 × 10^−6^
SKIV2L	15	1.88 × 10^−4^	rs389512	3.20 × 10^−6^
STK19_1	15	1.88 × 10^−4^	rs389512	3.20 × 10^−6^
NELFE	15	1.91 × 10^−4^	rs389512	3.20 × 10^−6^
CFB	15	1.92 × 10^−4^	rs389512	3.20 × 10^−6^
DXO	15	2.17 × 10^−4^	rs389512	3.20 × 10^−6^
CYP21A1P	5	8.96 × 10^−4^	rs389512	3.20 × 10^−6^
CYP21A2_1	5	8.73 × 10^−4^	rs389512	3.20 × 10^−6^

Top SNP rs389512 appears for all the top gene list due to very high LD.
