# Supplementary material for: Fine mapping genetic associations between the HLA region and extremely high intelligence
Source: Sci Rep. 2017 Jan 24;7:41182. doi: 10.1038/srep41182 (PMC5259706; doi:10.1038/srep41182)
Supplement: Supplementary Material [file srep41182-s1.pdf]

Supplementary Material

Fine mapping genetic associations between the HLA region and extremely high intelligence

Delilah Zabaneh[1], Eva Krapohl[1], Michael A. Simpson[2], Mike B. Miller[3], William G. Iacono[3], Matt McGue[3], Martha Putallaz[4], David Lubinski[5], Robert Plomin[1], Gerome Breen[1]

[1] MRC Social, Genetic and Developmental Psychiatry Centre, Institute of Psychiatry, Psychology & Neuroscience, King's College London, London SE5 8AF, UK.
[2] Division of Genetics and Molecular Medicine, Guy's Hospital, Great Maze Pond, London, SE1 9RT
[3] Department of Psychology, University of Minnesota, Minneapolis, MN 55455, USA.
[4] Duke University talent Identification Program, Duke University, Durham, NC 27701, USA.
[5] Department of Psychology and Human Development, Vanderbilt University, Nashville, TN 37203, USA

Correspondence to:

gerome.breen@kcl.ac.uk

Supplementary text

Extension sample: TEDS genotyping protocol, quality control and g-16 phenotype

TEDS is a longitudinal UK-based population sample of over 15 000 families with twins born in England and Wales between 1994, 1995 and 1996 and identified from birth records[1].

DNA for 4,649 indivdiuals was extracted from saliva and buccal cheek swab samples and hybridized to HumanOmniExpressExome-8v1.2 genotyping arrays at the Institute of Psychiatry, Psychology and Neuroscience Genomics & Biomarker Core Facility. The raw image data from the array were normalised, pre-processed, and filtered in GenomeStudio according to Illumina Exome Chip SOP v1.4.
(http://confluence.brc.iop.kcl.ac.uk:8090/display/PUB/Production+Version%3A+Illumina+E

xome+Chip+SOP+v1.4). In addition, prior to genotype calling 869 multi-mapping SNPs and 353 samples with callrate <.95 were removed. The ZCALL program (see Web resources section) was used to augment the genotype calling for samples and SNPs that passed the initial QC.

DNA from 3,665 samples was extracted from buccal cheek swabs and genotyped at Affymetrix, Santa Clara, California, USA. Samples were successfully hybridized to AffymetrixGeneChip 6.0 SNP genotyping arrays (http://www.affymetrix.com/support/technical/datasheets/genomewide_snp6_datasheet.pdf) using experimental protocols recommended by the manufacturer (Affymetrix Inc., Santa Clara, CA). The raw image data from the arrays were normalized and pre-processed at the Wellcome Trust Sanger Institute, Hinxton, UK for genotyping as part of the Wellcome Trust Case Control Consortium 2 (https://www.wtccc.org.uk/ccc2/) according to the manufacturer's guidelines (http://www.affymetrix.com/support/downloads/manuals/genomewidesnp6_manual.pdf). Genotypes for the Affymetrix arrays were called using CHIAMO (https://mathgen.stats.ox.ac.uk/genetics_software/chiamo/chiamo.html).

After initial quality control and genotype calling, the same quality control was performed on the samples genotyped on the Illumina and Affymetrix arrays separately using PLINK[2,3], R[4], and vcftools[5].

Samples were removed from subsequent analyses on the basis of call rate (<0.99), suspected non-European ancestry, heterozygosity, array signal intensity, and relatedness. SNPs were excluded if the minor allele frequency was <.05%, if more than 1% of genotype data were

missing, or if the Hardy Weinberg *p*-value was lower than 10^{-5}. Non-autosomal markers and indels were removed. Association between the SNP and the array, batch, or plate on which samples were genotyped was calculated; SNPs with an effect *p*-value less than 10^{-3} were excluded. A total sample of 6,710 samples, with 3,617 individuals and 600,034 SNPs genotyped on Illumina and 3,093 individuals and 525,859 SNPs genotyped on Affymetrix remained after quality control.

Genotypes from the two arrays were separately imputed using the Haplotype Reference Consortium [6] and Minimac3 1.0.13 [7,8] available on the *Michigan Imputation Server* as reference data. A series of quality checks was performed before merging data from the two arrays imputation (e.g. array effects, allele frequencies by imputation quality). For the present analyses we limited our analyses to variants genotyped or imputed at info >.70 on both arrays, allele frequency difference between arrays smaller than 5%, and Hardy Weinberg *p*-value was greater than 10^{-5}. Using these criteria, 7,581,516 genotyped and well-imputed SNPs were retained for the analyses.

We performed Principal Component Analysis on a subset of 42,859 common (MAF>5%) autosomal HapMap3 SNPs [9], after stringent pruning to remove markers in high linkage disequilibrium ($r^2 > 0.1$) and excluding high linkage disequilibrium genomic regions so as to ensure that only genome-wide effects were detected.

TEDS g-16 Phenotype

Individuals were tested at 12 years using, and g-16 was calculated using 16 tests and adjusting for age within each testing period, and first principal component scores were

derived using principal component analysis implemented in R. Imputed genotypes were available for 6710 samples.

Supplementary Tables

Table S1. Number of classical HLA alleles and amino acid residues after imputation using SNP2HLA

**Amino acid**	**HLA_A**	150	
	**HLA_B**	220	
	**HLA_C**	125	
**Amino acid**	**DPA1**	11	
	**DPB1**	33	
	**DQA1**	71	
	**DQB1**	134	
	**DRB1**	228	
		**2-digit**	**4-digit**
**Classical alleles**	HLA_A	13	11
	HLA_B	15	18
	HLA_C	12	15
	DPA1	2	2
	DPB1	8	8
	DQA1	5	7
	DQB1	5	12
	DRB1	12	13

Table S2. Power of HLA-wide association in the extreme sample using different assumptions of variance explained R calculated using GPC [10]

	**Effect size of single SNP on IQ (in R^2)**				
	**0.02%**	**0.04%**	**0.06%**	**0.08%**	**0.10%**
**HLA-wide (α= 8.7 x 10^{-6})**	0.02%	12%	33%	57%	77%
**Effective size (α =1.1 x 10^{-4})**	0.06%	27%	56%	78%	90%

Table S3. Summary results for the adjusted logistic regression model(1), of SNPs that reached significance with $P < 1.1 \times 10^{-3}$

SNP	BP	r^2 with rs444921	Ref allele	N	OR	SE	P	Location	Gene	TIP-MAF	CON-MAF
rs2523477	31468368	-	G	4662	0.64	0.12	1.08×10^{-4}	intergenic	HLA-B,MICA	0.09	0.07
rs2516472	31502679	-	C	4662	0.63	0.12	1.29×10^{-4}	intergenic	MICA,HCP5	0.08	0.07
rs9267659	31954213	-	A	4662	1.36	0.08	1.93×10^{-4}	intronic	SLC44A4	0.2	0.21
rs2523691	31528666	-	T	4662	0.64	0.12	2.21×10^{-4}	intergenic	MICA,HCP5	0.08	0.07
rs2596460	31525489	-	C	4662	0.64	0.12	2.28×10^{-4}	intergenic	MICA,HCP5	0.08	0.07
rs2596480	31533964	-	A	4662	0.64	0.12	2.28×10^{-4}	intergenic	HCP5	0.08	0.07
rs2523632	31453823	-	T	4662	0.65	0.12	2.40×10^{-4}	intergenic	HLA-B,MICA	0.08	0.07
rs2523639	31452077	-	A	4662	0.65	0.12	2.40×10^{-4}	intergenic	HLA-B,MICA	0.08	0.07
rs2507980	31455856	-	T	4662	0.65	0.12	2.44×10^{-4}	intergenic	HLA-B,MICA	0.08	0.07
rs2853972	31462323	-	T	4662	0.65	0.12	2.53×10^{-4}	intergenic	MICA	0.08	0.07
rs2844523	31476567	-	T	4662	0.65	0.12	2.79×10^{-4}	intronic	MICA	0.08	0.07
rs2523626	31454469	-	T	4662	0.65	0.12	2.94×10^{-4}	intergenic	HLA-B,MICA	0.09	0.07
rs2523547	31458133	-	A	4662	0.65	0.12	2.99×10^{-4}	intergenic	HLA-B,MICA	0.09	0.07
SNP_B_31431212	31431212	-	G	4662	0.81	0.06	3.48×10^{-4}	exonic	HLA-B	0.41	0.41

(1)Logistic model with sex and the first 10 principal components as covariates.

Table S4 Association for rs444921 and rs389512 in different studies.

Study	Pubmed ID	SNP	Aff allele	Beta	SE	P	MAF
**TEDS**	-	rs444921	-	-	-	-	-
		rs389512	C	0.0200	0.0103	0.53	-
**CHIC**[11]	23358156	rs444921	T	-0.0004	0.0194	0.98	0.09
		rs389512	-	-	-	-	-
**CHARGE**[12]	25201988	rs444921	T	0.0020	0.0082	0.80	-
		rs389512	C	0.0029	0.0083	0.73	-
**EA2**[13]	27225129	rs444921	-	-	-	-	-
		rs389512	C	-0.0018	0.0047	0.69	0.12
**VNR-UKB**[14]	27046643	rs444921	T	0.0123	0.0109	0.26	-
		rs389512	C	0.0123	0.0109	0.26	-
**Minnesota- IQ**	-	rs444921	T	-0.3013	0.5370	0.58	0.13
		rs389512	C	-0.2871	0.5351	0.59	0.13

Table S5. Neuropsychiatric traits associated with HLA variants reported in the GWAS catalogue [15]

Trait	Pubmed ID	Gene
Alzheimer's disease (late onset)	41745	HLA-DRB1 - HLA-DQA1
Autism spectrum disorder, attention deficit-hyperactivity disorder, bipolar disorder, major depressive disorder, and schizophrenia (combined)	41431	HLA-DQB1 - LOC102725019
Bipolar disorder and schizophrenia	41135	HLA-K - HLA-A
Dementia and core Alzheimer's disease neuropathologic changes	42213	HLA-DRA - HLA-DRB5
Narcolepsy	40443	LOC102725019 - HLA-DQA2
Narcolepsy (age of onset)	41772	HLA-DQB1
Narcolepsy (onset before 2009 H1N1 influenza pandemic)	41772	HLA-DRB1 - HLA-DQA1
Neuritic plaque	42213	-
Parkinson's disease	42133	HLA-DQB1 - LOC102725019
Schizophrenia	41325	HLA-DRB1 - HLA-DQA1

Table S6 Association for DRB1_08 and DRB1_11 alleles in our high cognition case-control data

Variant	Allele (A/P)	INFO	MAF	OR	SE	P
**HLA_DRB1_0801**	P	0.97	0.03	0.67	0.158	9.77×10^{-3}
**HLA_DRB1_1101**	P	0.80	0.06	1.09	0.075	2.55×10^{-1}

Figure S1. Forest plots for the meta-analysis of TIP-GWAS for top SNP rs444921 with a) all six studies and b) excluding the Minnesota study. SNP rs389512 was used as a proxy for rs444921 for study EA2.

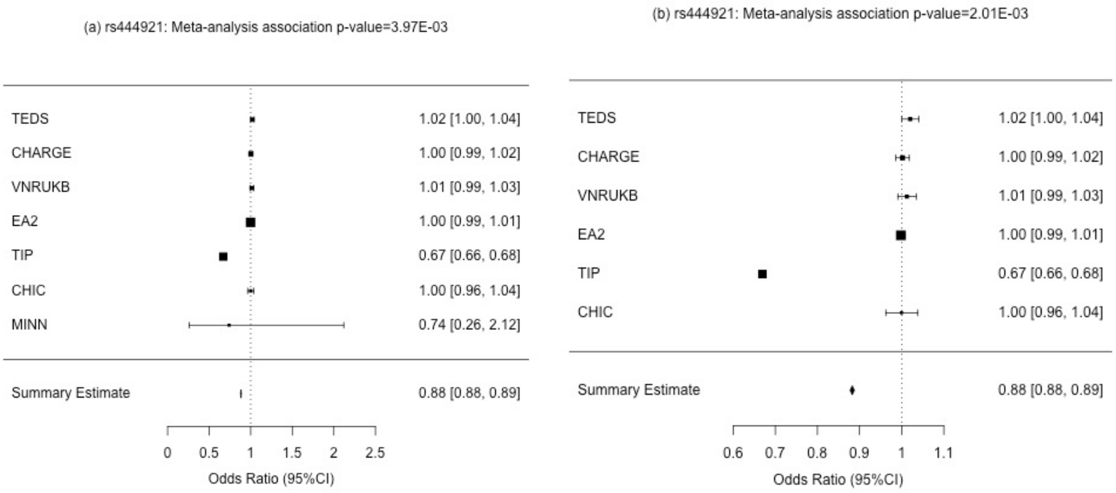

References

1. Haworth, C. M. A., Davis, O. S. P. & Plomin, R. Twins Early Development Study (TEDS): a genetically sensitive investigation of cognitive and behavioral development from childhood to young adulthood. *Twin Res. Hum. Genet.* **16,** 117–25 (2013).
2. Chang, C. C. *et al.* Second-generation PLINK: rising to the challenge of larger and richer datasets. *Gigascience* **4,** 7 (2015).
3. Purcell, S. *et al.* PLINK: a tool set for whole-genome association and population-based linkage analyses. *Am. J. Hum. Genet.* **81,** 559–75 (2007).
4. *R: A Language and Environment for Statistical Computing*. (R Foundation for Statistical Computing, 2015).
5. Danecek, P. *et al.* The variant call format and VCFtools. *Bioinformatics* **27,** 2156–2158 (2011).
6. McCarthy, S. *et al.* A reference panel of 64,976 haplotypes for genotype imputation. *bioRxiv* 35170 (2015). doi:10.1101/035170
7. Howie, B., Fuchsberger, C., Stephens, M., Marchini, J. & Abecasis, G. R. Fast and accurate genotype imputation in genome-wide association studies through pre-phasing. *Nat. Genet.* **44,** 955–959 (2012).
8. Fuchsberger, C., Abecasis, G. R. & Hinds, D. A. minimac2: faster genotype imputation. *Bioinformatics* **31,** 782–784 (2015).
9. Consortium, T. I. H. 3. Integrating common and rare genetic variation in diverse human populations. *Nature* **467,** 52–58 (2010).
10. Purcell, S., Cherny, S. S. & Sham, P. C. Genetic Power Calculator: design of linkage and association genetic mapping studies of complex traits. *Bioinformatics* **19,** 149–50 (2003).
11. Benyamin, B. *et al.* Childhood intelligence is heritable, highly polygenic and associated with FNBP1L. *Mol. Psychiatry* **19,** 253–258 (2014).
12. Rietveld, C. A. *et al.* Common genetic variants associated with cognitive performance identified using the proxy-phenotype method. *Proc. Natl. Acad. Sci.* **111,** 13790–13794 (2014).

13. Okbay, A. *et al.* Genome-wide association study identifies 74 loci associated with educational attainment. *Nature* **533,** 539–542 (2016).

14. Davies, G. *et al.* Genome-wide association study of cognitive functions and educational attainment in UK Biobank (N=112 151). *Mol. Psychiatry* **21,** 758–767 (2016).

15. Burdett, T. *et al.* The NHGRI GWAS Catalog. Available at: www.ebi.ac.uk/gwas. (Accessed: 26th July 2016)
